# First Reported Case of Reverse Pott's Disease

**DOI:** 10.1155/2022/3527704

**Published:** 2022-05-23

**Authors:** Lintu Ramachandran, David Lyu, Luqman Baloch, Mario Affinati

**Affiliations:** Department of Internal Medicine, Javon Bea Hospital, Rockford, IL, USA

## Abstract

Tuberculosis, while rare, is a disease that can have several extrapulmonary manifestations. One such known extrapulmonary manifestation of disseminated TB is vertebral osteomyelitis, often referred to as “Pott's Disease.” We present the case of a patient who underwent back surgery with allogenic bone graft who developed vertebral osteomyelitis and subsequently had disseminated tuberculosis, from an infected bone graft. We review current guidelines for allograft tissue screening and discuss the possible need for standardizing tuberculosis screening for bone allografts.

## 1. Introduction

A total of 7163 tuberculosis (TB) cases were reported in 2020 in the USA, of which 29% were in US-born persons [1]. Extrapulmonary TB is rare and is estimated to happen in 15% of all TB patients. Meningeal involvement in TB is even more uncommon with an estimated incidence of 5.4% of all extrapulmonary TB cases [2]. While primarily transmitted through aerosolization of respiratory droplets via cough, coughing is not mandatory for transmission. Any respiratory activity has been shown to increase risk of transmission [3]. We report the first case of a patient who underwent allograft bone graft placement with lumbar fusion and developed disseminated TB from contaminated bone graft.

## 2. Case Presentation

**Figure 1:**
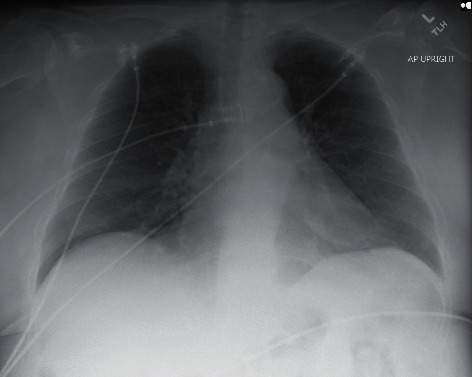
Chest X-ray of our patient showing diffuse nodular involvement of the lungs.

**Figure 2:**
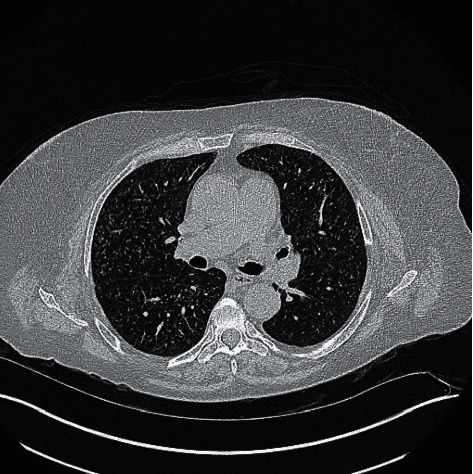
CT scan axial view showing conforming military tuberculosis with numerous 1–3 mm punctuate nodules.

Patient is a 66-year-old female with a medical history of peripheral arterial disease status after prior left common iliac stenting and neurogenic claudication secondary to L3-L4 spinal stenosis who presented to the hospital for generalized weakness, fatigue, and headache for 3 weeks. She also complained of intermittent back pain, fever, chills, and night sweats. She described new onset migraine like headache with photophobia in the last month. She denied coughing, shortness of breath, history of headaches, or any other new symptoms. She recently had minimally invasive L3–L5 lumbar fusion surgery with allograft bone graft placement 4 months ago. Her family history was unremarkable. Her social history was positive for 20 pack years of prior tobacco abuse. She quit smoking 2 years ago. She denied any alcohol or recreational drug use. On admission, her vitals including temperature were unremarkable. Her physical showed that patient was oriented to person and place, but not time. She was aware of her reasoning for admission but could not answer all questions appropriately. Healed incisions without any erythema, pus, or drainage were noted in the lower back at the L3–L5 level. Cranial nerves II–XII were grossly intact. Bilateral lower extremity strength was 4/5, unchanged from before, and no other focal deficits were noted. Laboratory evaluation was significant for sodium level of 117 mmol/L from 129 mmol/L two months prior, chloride of 77 mmol/L, and elevated platelet count of 425,000/*μ*L. ESR and CRP were elevated at >130 mm/h and 61.8 mg/L, respectively. TSH was normal at 2.4 mIU/mL. The random cortisol level was elevated at 29.5 *μ*g/dL. Serum osmolality was 256 mOsm/Kg. Urine osmolality was 487 mOsm/kg. Urine sodium and potassium were 32 mmol/L and 47 mmol/L, respectively. Urine toxicology and HIV test were negative. At this time, the treatment team and neurosurgeon who performed the lumbar surgery were informed by the Center for Disease Control (CDC) that the bone graft used in her surgery was contaminated with *Mycobacterium tuberculosis*. A chest X-ray (Figure 1) was done and showed diffuse miliary nodular pattern of both the lungs, which was confirmed by the presence of innumerable micronodules on CT chest (Figure 2).

MRI of the brain with contrast did not show any evidence of hydrocephalus, space-occupying lesions, or basilar meningitis. A lumbar puncture was done, and the results are given in Table 1.

**Figure 3:**
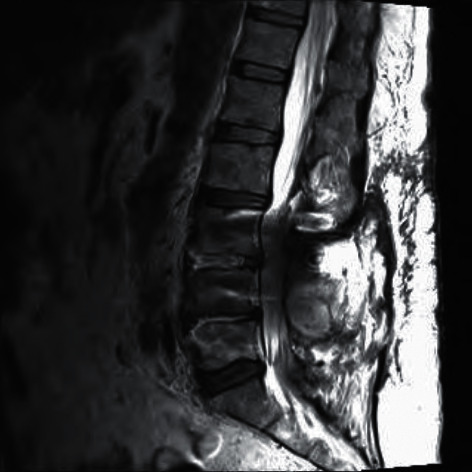
MRI of the lumbar spine revealing osteomyelitis at the L4-L5 level.

MRI of the lumbar spine showed L4-L5 osteomyelitis and postsurgical changes (Figure 3).

MRI of the cervical and thoracic spine was negative for any structural lesions. Due to the lack of cord compression symptoms and no evidence of compression of the thecal sac of the lumbar cistern, leading to severe stenosis, the risk of reoperating was deemed to be higher than any benefit. The decision was made between the infectious disease team and neurosurgery team to treat the patient medically and not have any further neurosurgical intervention. The patient was treated for disseminated TB from the graft infection with rifampin, isoniazid, pyrazinamide, and ethambutol. Ethambutol was switched to levofloxacin. Patient's mentation and headache improved gradually. Patient was discharged with plan for 12 weeks of total antituberculosis TB therapy. At 3-month follow-up, patient was noted to be doing well, and her symptoms, including back pain, headache, photophobia, encephalopathy, and weakness, had completely resolved. A repeat MRI of the lumbar spine with and without contrast did not show any further osteomyelitis.

## 3. Discussion

More than 500,000 bone graft procedures are performed in the United States annually [4]. Autogenous bone graft, made using the patient's own bone, is most commonly used. However, due to difficulty in obtaining autogenous bone grafts, their use is often limited. A commonly used viable alternative is allogenous bone grafts. Allografts are obtained from qualified living or recently deceased human donors and are further processed in tissue banks. Tissue banks have patented processes to remove almost 99% of bone marrow blood elements from the internal blood matrix [5]. Allogenic grafts, however, carry the risk of rejection and infection.

The exact prevalence of allograft-associated bone graft infection is unknown. A single center allogenic bone bank study conducted in 2005 revealed infection rate of 1.7% [6]. However, updates in screening and bone graft testing have likely reduced the infection rate further. The American Association of Tissue Banks (AATB) and U.S. Food and Drug Administration (FDA) have standardized screening and the testing procedure. Currently, all allograft samples must be collected aseptically in clean rooms. The harvested allograft samples then undergo further microbiological evaluation with HIV testing (antibodies as well as nucleic acid amplification test), hepatitis B (surface antigen, total antibodies to hepatitis B core antigen, and nucleic acid amplification testing), hepatitis C (antibodies and nucleic acid amplification testing), and syphilis (treponemal or treponemal test) [7].

TB testing is, however, not included in the guidelines for allografts, and as such, our patient's allograft was not tested for TB. Our patient had not travelled outside the United States and did not have any known risk factors for tuberculosis, such as prior incarceration or intravenous drug use. She obtained the infection from a contaminated lot of bone repair product, and several other patients from multiple states were infected, as was confirmed by CDC [8]. In Pott's disease, patients typically acquire spinal TB from a primary site of infection such as the lungs [9]. Our patient primarily had spinal TB from the bone graft and then subsequently developed disseminated TB, involving the lungs, representing the first case of “Reverse Pott's Disease.”

Our patient also developed hyponatremia, likely from syndrome of inappropriate antidiuretic hormone (SIADH) secondary to her TB infection. Hyponatremia is seen in 51% of patients with pulmonary TB [10]. SIADH has been linked to TB, and subsequent hyponatremia with reports dates back to 1965 [11]. Hyponatremia also has a similar prevalence (45%) in TB meningitis patients [12]. In TB meningitis cases, both SIADH and cerebral salt wasting have been noted to be the cause of hyponatremia [13].

Another plausible explanation for hyponatremia is adrenal tuberculosis. Our patient did not have hypotension or associated hyperkalemia. Her cortisol level was appropriately elevated at 30 *μ*g/dL. Our patient's CT abdomen/pelvis was negative for adrenal pathology. Moreover, TB-associated adrenalitis typically presents after almost 90% of the adrenals are destroyed and can take 2–4 years following disease onset [14]. Our patient's sodium levels also improved from 117 mmol/L to 126 mmol/L gradually with fluid restriction. In summary, our patient's elevated cortisol levels and lack of hypotension and hyperkalemia makes adrenal TB unlikely and points to SIADH as the likely culprit.

Our patient was successfully treated with rifampin, isoniazid, pyrazinamide, and levofloxacin. Levofloxacin was used instead of ethambutol due to better bone and CSF penetration [15]. She was also treated with dexamethasone for her meningitis. All hospital partners and family members who encountered the patient were tested for TB as per CDC recommendations. Our case highlights the potential benefit of standardizing TB testing in allografts. A study published in 2007 reviewed 15 years of data from 27840 allograft heart valve tissues. These allografts were stained for TB, but all were negative [16]. The cost associated with tuberculosis treatment, however, per patient in the USA is estimated to be $17,000 (non-multidrug-resistant TB), $134,000 (multidrug-resistant TB), and $430,000 (extensively drug-resistant TB) [17]. The cost versus benefit analysis of allograft TB testing is an area that certainly needs more investigation.

## 4. Conclusion

In conclusion, we present the first case of disseminated TB infection from a contaminated bone graft. Eventhough the incidence of TB from bone graft is low, the high morbidity and mortality associated with TB, the cost of treatment, and the high contagion risk in the event of a potential TB infection make standardizing allograft TB screening a notion that needs further research and discussion.

## Figures and Tables

**Table 1:** Lumbar puncture results of our patient.

	Normal	Bacterial	Viral	TB	Our patient
WBC count	0–5/mm^3^	100–20,000/mm^3^	5–500/mm^3^	5–2000/mm^3^	408/mm^3^
WBC predominance	None	Neutrophilic	Lymphocytic	Lymphocytic	Lymphocytic
Protein concentration	15–50	100–500 mg/dL	<150 mg/dL	>50 mg/dL	93 mg/dL
Glucose	45–100 mg/dL	<40 mg/dL	30–70 mg/dL	<40 mg/dL	19 mg/dL
